# The prescription pattern and awareness about antibiotic prophylaxis and resistance among a group of Egyptian pediatric and general dentists: a cross sectional study

**DOI:** 10.1186/s12903-021-01685-y

**Published:** 2021-06-26

**Authors:** Mariam Mohsen Aly, Marwa Aly Elchaghaby

**Affiliations:** Pediatric Dentistry and Dental Public Health, Faculty of Dentistry, Cairo University, Giza, Egypt

**Keywords:** Antibiotics, Awareness, Prescription, Prophylaxis, Resistance

## Abstract

**Background:**

The use of antibiotics in dentistry as prophylaxis and treatment is frequent. Their misuse has led to a major public health problem globally known as antibiotic resistance. This study aimed to assess the pattern of antibiotic prescription and its prophylactic use for systemic conditions. Besides, this study evaluated the awareness and adherence to antibiotic prescription guidelines and antibiotic prophylaxis guidelines along with awareness of antibiotic resistance across pediatric and general dentists.

**Methods:**

An overall of 378 pediatric and general dentists meeting the required eligibility criteria, fulfilled a pre-designed validated questionnaire. Data were collected, tabulated, and statistically analyzed.

**Results:**

A significant statistical difference was found among the pediatric and general dentists regarding antibiotics prescription for most of the oral conditions where Amoxicillin with clavulanic acid was the most frequently prescribed antibiotic among the two groups (53% pediatric dentist and 52% general dentist). The majority of pediatric and general dentists, on the other hand, were aware of antibiotic resistance and prescribing recommendations.

**Conclusions:**

The present study showed a tendency to overprescribe and overuse antibiotics in certain dental conditions among the participants. The vast majority of dentists, especially general dentists do not have adherence to professional guidelines for antibiotics prescription in children despite their awareness of antibiotic resistance and prescription guidelines.

**Supplementary Information:**

The online version contains supplementary material available at 10.1186/s12903-021-01685-y.

## Background

Antibiotics have been widely used in children and adults since Alexander Fleming discovered penicillin in 1928 and Howard Walter Florey began using it clinically in 1941. Dentists routinely prescribe antibiotics for therapeutic or prophylactic reasons for the management of oral and dental infections [1, 2].

From 2000 to 2015, the global antibiotic consumption in terms of defined daily doses has seen an increase of 65%, mainly in low middle-income countries where the use of antibiotics in developing countries has been observed to be much higher compared to the developed world [3].

The success of antibiotics has been absolute, with a worldwide enhancement in morbidity and mortality. Though, this early success is being battered by the microbial capability to grow and renovate to protect themselves, thus neutralizing the therapeutic effects of antibiotics [4].

In the twenty-first century, the increasing emergence of antibiotic-resistant bacteria is evolving into a major public health threat. This threat is no longer a prediction for the future but a current finding in every region of the world, claiming 700,000 lives each year, a figure that could rise to 10 million by 2050 [3, 5].

Although there are lots of causes for the increased rates of resistance, the overuse of antibiotics is the most critical, even though the antibiotic prescription is still deemed an insignificant act [6, 7]. Unsuitable use of broad-spectrum antibiotics, wrong selection of drugs, and incorrect dose or duration were also reported. Patients also participate by not taking antibiotics as recommended, such as missing doses or ceasing earlier than advised [5, 7].

It was stated in 2015, that 40–50% of antibiotic prescriptions worldwide were unnecessary [6]. The proportion of antibiotics prescribed by dental practitioners comprises around 3%–11% of all prescribed antibiotics, making the contribution of dentists to antibiotic consumption significant [8, 9].

A contribution from pediatric dentists (PD) has also been noted as an unwarranted use of antibiotics and was reported in children with orofacial infections [3, 10]. Surveys have also indicated that the awareness of antibiotic resistance and the impact of antibiotic prescribing by dentists are low [8].

The observed prescribing behavior in reference to pediatric dentistry could be a result of the pressure of the parent where this parental pressure has been reported as one of the primary causes of unwarranted prescriptions. Other non-clinical situations acting as determinants for antibiotic use include uncertain diagnosis of the case, need for delay of treatment owing to unavailable appointments, problems like ineffective sterilization, and social relations. Also, it was suggested that practitioners assume that using antibiotics is the quickest way to resolve any form of consultation [10].

Many studies in the literature described general and pediatric dentists’ antibiotic prescription patterns worldwide [11–14]. However, to the best of our knowledge, there is no data available describing Egyptian dentists’ general practices, and very few studies explored dentists’ attitudes regarding antibiotic use and resistance. Therefore, the present study aimed to assess the pattern of antibiotic prescription and its prophylactic use for systemic conditions. Besides, this study evaluated the awareness and adherence to antibiotic prescription guidelines and antibiotic prophylaxis guidelines along with awareness of antibiotic resistance across pediatric and general dentists (GD).

## Methods

### Study design

This was a cross-sectional questionnaire study conducted among a random sample of Egyptian dentists, with an electronic link to the questionnaire produced by Google forms and distributed via email. The STROBE guidelines were used to ensure the reporting of this observational study.

### Ethical aspects

The current research was carried out in compliance with the Helsinki Declaration. Ethical approval was obtained from the Ethics Committee of Scientific Research, Faculty of Dentistry, Cairo University. Dentists were notified about the objective of the study and informed consent was acquired at the beginning of participation to answer the online survey questionnaire with the maintenance of confidentiality and anonymity by the settings of the online survey.

### Sample size

According to the results of Konde et al. [12] where the frequency of pediatric dentists aware of the prescription guidelines of antibiotics and antibiotic resistance was (56%), by adopting a confidence interval of (95%), the predicted sample size (n) was a total of (378) participants using Epi info program.

### Participants

#### Inclusion criteria

- General dentists holding a Bachelor of oral and Dental medicine or a master's or doctorate in a specialty other than pediatric dentistry.
- Pediatric dentists holding a master's or doctorate.

#### Exclusion criteria

- Any nationality except Egyptian.
- Dentists who aren't engaged in clinical practice.
- Dentists who didn't treat children.

### Outcomes

This survey aimed to assess:

- The antibiotic prescription patterns in case of pulpitis, draining sinus tract, localized intraoral swelling, acute facial swelling, dental trauma, pediatric periodontal diseases, pericoronitis, simple extraction, extraction by open method, periapical abscess, apical periodontitis, dry socket, evidence of anaerobic infection in children.
- Use of antibiotics as prophylaxis for systemic conditions including cardiovascular diseases, viral infections, juvenile diabetes, blood dyscrasias, respiratory disorders in children.
- The awareness and adherence to the antibiotic prescription guidelines.
- The awareness and adherence to guidelines for antibiotic prophylaxis.
- The awareness of antibiotic resistance across pediatric and general dentists.
- The dentist's perspective of possible causes of antibiotic misuse.

### Data sources and measurement

An English pre-prepared validated self-administered questionnaire based on the previous work of Konde et al. [12] after taking their permission, was used to collect data (Additional file 1).

An electronic link to the questionnaire was generated using Google forms and was distributed to a representative sample of 1512 dentists through emails. The study sample was chosen using a random number generator from a list containing all dentists fulfilling the inclusion and exclusion criteria obtained from the official registry of the Faculty of Dentistry, Cairo University.

The questionnaire was prefaced with the consent section which explained the study's purpose, nature of the survey, study objectives, and voluntary participation. Informed consent was obtained from each participant in the form of answering a question (Yes/No) before proceeding with answering the questionnaire.

The questionnaire consisted of close-ended questions (Yes/No) or multiple choices. Four questions related to demographic details and practice information including specialty, graduation university, clinical experience, and work practice.

Twenty questions describing clinical situations for which antibiotics were prescribed routinely, the most commonly prescribed antibiotics, the duration of the antibiotic course, prescription in presence of anaerobic infection, and systemic conditions.

Along with awareness of antibiotic resistance, the awareness and adherence to the AAPD guidelines for use of antibiotic therapy for pediatric dental patients [15] and antibiotic prophylaxis guidelines including AAPD guideline for antibiotic prophylaxis for dental patients at risk for infection [16], and American Heart Association guideline for Prevention of Infective Endocarditis [17] were evaluated in five questions.

Possible causes of antibiotic misuse from the dentist's perspective were evaluated in six questions with Yes/No answers.

### Bias

All dental practitioners who took part in the study were chosen randomly and asked to complete a self-administered questionnaire anonymously to limit selection bias. All participants were given the same explanation about the study's nature and purpose to limit information bias [18].

### Statistical analysis

The statistical analysis program Statistical Package for Social Sciences (SPSS) version 22.0 was used to undertake all the statistical analysis. For the categorical data, descriptive statistics were used to calculate numbers, frequencies, and percentages for each category. A Chi-square test was utilized for comparing prescription patterns between general and pediatric dentists.

## Results

Out of 1512 mailed questionnaires, only a total of 378 dentists completed the questionnaire where 154 (40.7%) were pediatric dentists and 224 (59.3%) were general dentists with a 25% response rate. The demographic data and practice information of the participants were displayed in Table 1 where 55.6% of participants were graduated from public universities and 44.4% were graduated from private universities. About 36.5% of the study population had clinical experience for less than two years, 28.6% from 2 to 5 years, and 34.9% for more than 5 years while 23.0% of participants worked in clinical practices, 35.2% in academic institutions, and 41.8% in both clinical practices and academic institutions.

**Table 1:** Demographic data and practice information for the study population

Variable	Number (N)	Percentage (%)
Specialty		
Pediatric dentists (PD)	154	40.7
General dentists (GD)	224	59.3
Graduation university		
Public	210	55.6
Private	168	44.4
Working experience in years		
Less than 2 years	138	36.5
From 2 to 5 years	108	28.6
More than 5 years	132	34.9
Working place		
Clinical practice	87	23.0
Academics	133	35.2
Both	158	41.8

Clinical situations for routine antibiotic prescriptions were demonstrated in Table 2 where there was a significant statistical difference in the prescription of antibiotics among the pediatric dentists and general dentists for a number of the oral conditions except for periapical abscess, dry socket, pediatric periodontal diseases, pericoronitis, and extraction by the open method.

**Table 2:** Clinical situations for routine antibiotics prescription among pediatric dentists and general dentists

Clinical situations	Answer Yes/No	Pediatric dentists (N = 154)		General dentists (N = 224)		p-value
		Number (N)	Percentage (%)	Number (N)	Percentage (%)	
Pulpitis	Yes	1	0.6	10	4.5	0.030
	No	153	99.4	214	95.5	
Draining sinus tract	Yes	15	9.7	51	22.8	0.001
	No	139	90.3	173	77.2	
Localized intraoral swelling	Yes	20	13.0	53	23.7	0.010
	No	134	87.0	171	76.3	
Acute facial swelling	Yes	136	88.3	173	77.2	0.006
	No	18	11.7	51	22.8	
Dental trauma	Yes	76	49.4	70	31.3	0.000
	No	78	50.6	154	68.8	
Pediatric periodontal diseases	Yes	28	18.2	29	12.9	0.162
	No	126	81.8	195	87.1	
Pericoronitis	Yes	69	44.8	92	41.1	0.471
	No	85	55.2	132	58.9	
Simple extraction	Yes	0	0	6	2.7	0.041
	No	154	100	218	97.3	
Extraction by open method	Yes	59	38.3	83	37.1	0.804
	No	95	61.7	141	62.9	
Periapical abscess	Yes	90	58.4	141	62.9	0.377
	No	64	41.6	83	37.1	
Apical periodontitis	Yes	41	26.6	35	15.6	0.009
	No	113	73.4	189	84.4	
Dry socket	Yes	48	31.2	61	27.2	0.406
	No	106	68.8	163	72.8	
Evidence of anaerobic infection	Yes	134	87.0	170	75.9	0.007
	No	20	13.0	54	24.1	

The antibiotic prescription patterns among the pediatric and general dentists were displayed in Table 3 where Amoxicillin with clavulanic acid was the most commonly prescribed antibiotic (53% PD and 52% GD) followed by Amoxicillin (32% PD and 27% GD). Regarding the duration of the antibiotic course, the majority of the dentists (90% PD and 86% GD) prescribed antibiotics for 5–7 days as demonstrated in Table 4. However in the evidence of anaerobic infection, pediatric dentists' prescriptions varied in comparison to the general dentists with a statistical significance (p = 0.007) as revealed in Table 2.

**Table 3:** The most commonly prescribed antibiotic in pediatric and general dentists

The most commonly prescribed antibiotic	Pediatric dentists (N = 154)		General dentists (N = 224)		p-value
	Number (N)	Percentage (%)	Number (N)	Percentage (%)	
Amoxicillin	50	32.5	62	27.7	0.316
Amoxicillin with clavulanic acid	83	53.9	118	52.7	0.816
Ampicillin with Sulbactam	2	1.3	3	1.3	0.973
Cephalosporins	2	1.3	8	3.6	0.176
Clindamycin	5	3.2	21	9.4	0.021
Amoxicillin with flucloxacillin	12	7.8	12	5.4	0.340

**Table 4:** Duration of the antibiotic course among pediatric and general dentists

Duration of the antibiotic course	Pediatric dentists (N = 154)		General dentists (N = 224)		P-value
	Number (N)	Percentage (%)	Number (N)	Percentage (%)	
Less than 5 days	12	7.8	21	9.4	0.592
5 to 7 days	140	90.9	193	86.2	0.161
More than 7 days	2	1.3	10	4.5	0.085

A large number of pediatric dentists (90.3%) and general dentists (89.3%) prescribe prophylactic antibiotics in cases of cardiovascular diseases with no statistical significance between both groups (p = 0.760) while the majority of pediatric and general dentists didn’t prescribe antibiotics in case of viral infections, juvenile diabetes, blood dyscrasias, and respiratory disorders with no statistical significance between both groups as demonstrated in Table 5.

**Table 5:** Prescription of antibiotics for systemic conditions

Systemic conditions	Answer Yes/No	Pediatric dentists (N = 154)		General dentists (N = 224)		p-value
		Number (N)	Percentage (%)	Number (N)	Percentage (%)	
Cardiovascular diseases	Yes	139	90.3%	200	89.3%	0.760
	No	15	9.7	24	10.7%	
Viral infections	Yes	14	9.1%	18	8.0%	0.717
	No	140	90.9	206	92.0	
Juvenile diabetes	Yes	23	14.9%	27	12.1%	0.417
	No	131	85.1%	197	87.9	
Blood dyscrasias	Yes	16	10.4%	24	10.7%	0.920
	No	138	89.6	200	89.3	
Respiratory disorders	Yes	14	9.1%	23	10.3%	0.705
	No	140	90.9	201	89.7	

Concerning the awareness of the guidelines, 67.5% of pediatric dentists, and 62.1% of general dentists were aware of the antibiotic prescription guidelines with no statistical significance between both groups (p = 0.275) while 74.7% of pediatric dentists and 70.1% of general dentists were aware of the antibiotic prophylaxis guidelines with no statistical significance between both groups (p = 0.329) as displayed in Table 6.

**Table 6:** Awareness of pediatric and general dentists to the antibiotic prescription and prophylaxis guidelines, and antibiotic resistance

	Answer Yes/No	Pediatric dentists (N = 154)		General dentists (N = 224)		p-value
		Number (N)	Percentage (%)	Number (N)	Percentage (%)	
Awareness of the antibiotic prescription guidelines	Yes	104	67.5	139	62.1	0.275
	No	50	32.5	85	37.9	
Adherence to the antibiotic prescription guidelines	Yes	105	68.2	120	53.6	0.004
	No	49	31.8	104	46.4	
Awareness of the antibiotic prophylaxis guidelines	Yes	115	74.7	157	70.1	0.329
	No	39	25.3	67	29.9	
Adherence to antibiotic prophylaxis guidelines	Yes	105	68.2	165	73.7	0.247
	No	49	31.8	59	26.3	
Awareness of antibiotic resistance	Yes	153	99.4	216	96.4	0.067
	No	1	0.6	8	3.6	

Regarding adherence to guidelines, 68.2% of pediatric dentists and 53.6% of general dentists were adherents to the antibiotic prescription guidelines with statistical significance between both groups (p = 0.004) while 68.2% of pediatric dentists and 73.3% of general dentists were adherents to the antibiotic prophylaxis guidelines with no statistical significance between both groups (p = 0.247) as displayed in Table 6.

Nearly all pediatric and general dentists were aware of antibiotic resistance and most of them grasped that self-medication and injudicious use of antibiotics lead to the development of antibiotic resistance with no statistical significance between both groups as revealed in Table 6.

Before prescribing antibiotics, the majority of participants ask whether the patient has taken antibiotics in the previous week and instruct them to adhere to the dose as demonstrated in Table 7.

**Table 7:** The possible causes of antibiotic misuse from the dentist's perspective

Types	Reasons	Answer Yes/No	Pediatric dentists (N = 154)		General dentists (N = 224)		p-value
			Number (N)	Percentage (%)	Number (N)	Percentage (%)	
Patient-related	Self-medication	Yes	152	98.7	217	96.9	0.252
		No	2	1.3	7	3.1	
	Insisting parents	Yes	5	3.2	10	4.5	0.551
		No	149	96.8	214	95.5	
Dentist-related	Inquiring the patient about taking antibiotics in the past week before prescribing antibiotics	Yes	140	90.9	174	77.7	0.001
		No	14	9.1	50	22.3	
	Advising the patient to adhere to the dosage regimen and informing the consequences of not doing so	Yes	151	98.1	214	95.5	0.187
		No	3	1.9	10	4.5	
	Prescribing antibiotics if you have many appointments already waiting at your clinic	Yes	9	5.8	10	4.5	0.5462
		No	145	94.2	214	95.5	
	Prescribing antibiotics to sustain the patient until the specialist treats the patient	Yes	17	11.0	33	14.7	0.298
		No	137	89.0	191	85.3	

Only 5% of general and pediatric dentists prescribed antibiotics because of insisting parents or the presence of too many patients in the waiting area and less than 15% prescribed antibiotics to sustain the patient until the specialist treats the patient with no statistical significance between both groups as revealed in Table 7.

## Discussion

Antibiotic resistance is growing to alarmingly high levels worldwide, endangering our capability to treat frequent infectious diseases. This antibiotic resistance is exacerbated by antibiotic misuse and overuse, as well as ineffective infection prevention and control [19].

Reinforcement of the global antimicrobial resistance surveillance is crucial for establishing global strategies, monitoring the effectiveness of public health interventions, and detecting new trends and threats. Hence, This study is an attempt to contribute to the available literature on antibiotic use and abuse, particularly when treating children in the Middle East [13].

The present study showed a tendency to overprescribe and overuse antibiotics in certain conditions like pulpitis, draining sinus tract, localized intraoral swelling, periapical abscess, apical periodontitis, dry socket which occurred more by general dentists in comparison to pediatric dentists. These findings were in accordance with several studies which indicate that inadequate understanding of the disease, uncertain diagnosis, time pressure, patient expectation, parental pressure, and refusal of operative treatment may be the primary reasons [5, 7, 13, 20].

Amoxicillin with clavulanic acid was the most frequently prescribed antibiotic followed by Amoxicillin. It may be contributed to the effectiveness of Amoxicillin against Streptococci and oral anaerobes which make them appropriate for the treatment of odontogenic infections and the advantage of Amoxicillin with clavulanic acid to preserve the activity against the Beta-lactamases commonly produced by microorganisms associated with odontogenic infections [14, 21].

According to the American Academy of Pediatric Dentistry, Metronidazole may be prescribed as a supplementary antimicrobial treatment in the presence of anaerobic bacterial involvement [15]. This may explain the variation in antibiotic prescription in the presence of an anaerobic infection between pediatric and general dentists in the present study.

Regarding the duration of antibiotic prescription, the majority of the dentists in both groups prescribed antibiotics for 5–7 days allowing resolution of signs and symptoms with no risk of clinical and microbiological relapse. Moreover, antibiotic prescription for the correct duration lowers the unfavorable results and relieves the dilemma of antibiotic resistance [14, 15, 21].

Concerning antibiotic prescription for systemic conditions, a majority of dentists would prescribe antibiotics in cases of cardiovascular diseases while in case of viral infections, juvenile diabetes, blood dyscrasias, respiratory disorders majority of dentists declared they would not prescribe any antibiotics.

While there is a possibility that oral microorganisms can germ and infects distant tissues after oral practices, there is no proven evidence that this happens. Therefore, the fact of when and for which situations systemic prophylactic antibiotics are needed is debatable [21, 22].

The American Heart Association (AHA) proposes antibiotic prophylaxis for patients with cardiac disorders as they have the greatest risk of an unfavorable outcome [16, 17].

The awareness of guidelines for the prescription and prophylaxis of antibiotics was found to be around 70% of the study population where only 30% felt inadequately informed and trained regarding antibiotic use with no statistical significance between both groups in line with previous studies [12, 20].

Pediatric dentists showed comparatively better adherence to the guidelines as compared to general dentists in our study which may be contributed to the fact that pediatric dentists treated children more often and usually have more years of education in treating children [11].

But still, adherence of pediatric dentists to guidelines was considered low (less than 60%). This finding was in accordance with previous studies that have demonstrated varying low levels of adherence among pediatric dentists in various parts of the world, ranging from 10 to 56% [3, 11–13].

Despite the awareness of antibiotic resistance and prescription guidelines among most of the participants, still there is a misuse in antibiotics prescription. This might be explained by the fact that although knowledge of clinical guidelines and research evidence directly influence antibiotic prescribing, other barriers or competing factors exist that hinder their use in the oral health setting [23].

The results of the present study showed that the majority of the participants inquire the patient about taking antibiotics in the past week before prescribing antibiotics and advise them to adhere to the dosage regimen. Also, the presence of too many patients in the waiting areas or the need to sustain the patient till the next appointment were not reasons for prescribing antibiotics. Dentists demonstrate a lack of worry in limiting this major problem which may be defended by the statement of self-medication as a trigger for its advancement.

This may be explained by the socially desirable responding phenomena, that is, when inquired about unprofessional acts, participants may reply in a way that they feel socially acceptable rather than disclosing information about their true behavior [24].

### Study limitations

The data collected was self-reported by the participants and did not examine the actual prescription from the patient files. Furthermore, reporting bias is a concern, as dentists’ responses may not truly reflect their actual practice. In addition, non-response bias is another possible drawback of self-administered questionnaire research. Respondents' answers may have varied more positively or negatively from those of non-respondents, making it difficult to predict which direction the non-responders will lean.

## Conclusions

The present study showed a tendency to overprescribe and overuse antibiotics in certain dental conditions among the participants. The vast majority of dentists, especially general dentists do not have adherence to professional guidelines for antibiotics prescription in children despite their awareness of antibiotic resistance and prescription guidelines. Nearly all pediatric and general dentists were aware of antibiotic resistance, and the majority of them were also aware of the guidelines for the prescription and prophylactic use of antibiotics for systemic conditions.

## Supplementary Information

**Additional file 1.** Questionnaire used in the present study.

## Data Availability

The datasets used are available from the corresponding author on reasonable request.

## Abbreviations

PD: Pediatric dentists
GD: General dentists
