# Supplementary material for: The prescription pattern and awareness about antibiotic prophylaxis and resistance among a group of Egyptian pediatric and general dentists: a cross sectional study
Source: BMC Oral Health. 2021 Jun 26;21:322. doi: 10.1186/s12903-021-01685-y (PMC8235867; doi:10.1186/s12903-021-01685-y)
Supplement: Supplementary file 1 — Additional file 1. Questionnaire used in the present study. [file 12903_2021_1685_MOESM1_ESM.pdf]

Questionnaire

Specialty: a. Pediatric dentist b. General dentist

Graduation university: a. Public b. Private

Work experience in years: a. Less than 2 years
b. From 2 to 5 years
c. More than 5 years

Working place: a. Clinical practice
b. Academics
c. Both

Do you routinely prescribe antibiotics in the following situations?

- Pulpitis: a. Yes b. No
- Draining sinus tract: a. Yes b. No
- Localized intraoral swelling: a. Yes b. No
- Acute facial swelling: a. Yes b. No
- Dental trauma: a. Yes b. No
- Pediatric periodontal diseases: a. Yes b. No
- Pericoronitis: a. Yes b. No
- Simple extraction: a. Yes b. No
- Extraction by the open method:a. Yes b. No
- Periapical abscess: a. Yes b. No
- Apical periodontitis: a. Yes b. No
- Dry socket: a. Yes b. No
- Evidence of anaerobic infection: a. Yes b. No

Which is the most commonly prescribed antibiotic by you?

a. Amoxicillin
b. Amoxicillin with clavulanic acid
c. Ampicillin with Sulbactam
d. Cephalosporins
e. Clindamycin
f. Amoxicillin with flucloxacillin

What is the duration of the antibiotic course?

a. Less than 5 days
b. 5 to 7 days
c. More than 7 days

Do you prescribe antibiotics for the following systemic conditions:

a. Cardiovascular diseases: a. Yes b. No
b. Viral infections: a. Yes b. No
c. Juvenile diabetes: a. Yes b. No
d. Blood dyscrasias: a. Yes b. No
e. Respiratory disorders: a. Yes b. No

Are you aware of the current AAPD guidelines for "antibiotic prescription "? a. Yes b. No

Do you follow the same? a. Yes b. No

Are you aware of the current AAPD guidelines for "antibiotic prophylaxis"? a. Yes b. No

Do you follow the same? a. Yes b. No

Are you aware of the term "antibiotic resistance'?

a. Yes

b. No

Self-medication with antibiotics by patients to get relief from dental pain may be responsible for antibiotic misuse.

a. Yes

b. No

Do you prescribe antibiotics when the parents insist that the treatment should be completed soon?

a. Yes

b. No

Do you inquire from your patient about whether he/she has taken a course of antibiotics in the past 1 week before prescribing antibiotics?

a. Yes

b. No

Do you advise your patient to adhere to the dosage regimen and inform the consequences of not doing so?

a. Yes

b. No

Do you prescribe antibiotics if you have many appointments already waiting at your clinic?

a. Yes

b. No

Do you prescribe antibiotics to sustain the patient until the specialist treats the patient?

a. Yes

b. No
